# Adsorption and desorption of self-assembled L-cysteine monolayers on nanoporous gold monitored by in situ resistometry

**DOI:** 10.3762/bjnano.10.219

**Published:** 2019-11-18

**Authors:** Elisabeth Hengge, Eva-Maria Steyskal, Rupert Bachler, Alexander Dennig, Bernd Nidetzky, Roland Würschum

**Affiliations:** 1 Insitute of Materials Physics, Graz University of Technology, Petersgasse 16, A-8010 Graz, Austria; 2 Institute of Biotechnology and Biochemical Engineering, Graz University of Technology, Petersgasse 12, A-8010 Graz, Austria

**Keywords:** L-cysteine, in situ resistometry, nanoporous gold, self-assembled monolayer (SAM), voltammetry

## Abstract

Surface modifications of nanoporous metals have become a highly attractive research field as they exhibit great potential for various applications, especially in biotechnology. Using self-assembled monolayers is one of the most promising approaches to modify a gold surface. However, only few techniques are capable of characterizing the formation of these monolayers on porous substrates. Here, we present a method to in situ monitor the adsorption and desorption of self-assembled monolayers on nanoporous gold by resistometry, using cysteine as example. During the adsorption an overall relative change in resistance of 18% is detected, which occurs in three distinct stages. First, the cysteine molecules are adsorbed on the outer surface. In the second stage, they are adsorbed on the internal surfaces and in the last stage the reordering accompanied by additional adsorption takes place. The successful binding of cysteine on the Au surface was confirmed by cyclic voltammetry, which showed a significant decrease of the double-layer capacitance. Also, the electrochemically controlled desorption of cysteine was monitored by concomitant in situ resistometry. From the desorption peak related to the (111) surface of the structure, which is associated with a resistance change of 4.8%, an initial surface coverage of 0.48 monolayers of cysteine could be estimated.

## Findings

Nanoporous gold, produced by selective etching of the less noble component of a AuAg master alloy (also known as dealloying), is a very promising material in many applications due to its three-dimensional nanoporous structure. Among many other technological applications as sensing [1–2] and energy storage [3], nanoporous metals exhibit great potential in biochemical applications [4]. In addition to their various useful intrinsic characteristics, such as high surface-to-volume ratio and tunable mechanical properties, some applications require a modification of the surface, e.g., by using the well-known spontaneous formation of self-assembled monolayers (SAMs) on gold substrates [5]. These layers usually consist of three groups: a thiol as the head group, a carbon chain as the backbone and an end group that can be chosen according to the requirements for subsequent application. For planar Au surfaces, self-assembly processes are known to occur in adsorption and ordering (also known as “standing up”) stages [6]. The adsorption and ordering of such monolayers has been subject to experimental as well as theoretical investigation for many years, mostly studying the deposition on single crystalline surfaces. Using nanoporous substrates for modification with SAMs, however, is more complicated, as the applicable techniques for characterization are very limited, whereby electrochemical methods seem most promising [7–8].

Here, we present a new method to monitor and characterize the ad-/desorption of SAMs on nanoporous gold (npAu), based on our experience on in situ resistometry as a highly sensitive diagnosis tool for ad-/desorption processes [9]. The selected SAM material for this study is cysteine, due to its beneficial properties such as the short carbon backbone, the solubility in water and the easy handling.

The npAu substrate material was fabricated by electrochemically assisted dealloying of Ag_75_Au_25_. The alloy was prepared by arc melting, homogenized at 800 °C for 12 h under argon atmosphere, rolled to a sheet of 220 μm in thickness, annealed again at 600 °C for 1 h, and cut in rectangular pieces sized 10 × 5 mm. Two representative samples are presented here, referred to as sample A (91 mg) and sample B (86 mg). Given weights refer to the master alloy.

Each sample was contacted by five flattened Au wires, attached in line. The mid-positioned wire served as connection to an Autolab PGSTAT204 potentiostat, the others for four-point resistance measurements with a Keithley 2400 Source Meter. For a more detailed description of the resistometry setup, the reader is referred to our previous work [9]. An electrochemical cell was set up in three-electrode geometry, using a commercial Ag/AgCl reference electrode (saturated KCl with a 3 M KNO_3_ salt bridge), relative to which all potentials will be stated in the following. Whenever the cell electrolyte was changed, the setup was immersed in distilled water for several hours for rinsing.

Dealloying was performed in 0.1 M HClO_4_ with a Pt-wire counter electrode under chronoamperometric conditions at *U*_Ag/AgCl_ = 1100 mV until the current had fallen below 50 μA. For all following procedures, a carbon fabric served as the counter electrode. After dealloying, the sample was cleaned from the primary oxide [10] by cyclic voltammetry (CV) in 0.1 M HClO_4_ solution. The SAMs were deposited from a 20 mM cysteine solution (ROTH company, L-cysteine ≥98% in ultrapure water). Sample A was immersed directly into 20 mL of the cysteine solution, while sample B was first immersed in 10 mL ultrapure water, then 10 mL of 40 mM cysteine solution was added. Both samples were transferred directly from the rinsing solution, the different immersion procedures were chosen for detailed comparison of the resistance behavior caused by the cysteine molecules. The samples were exposed to cysteine solution for a total timespan of approximately 95 h.

**Figure 1:**
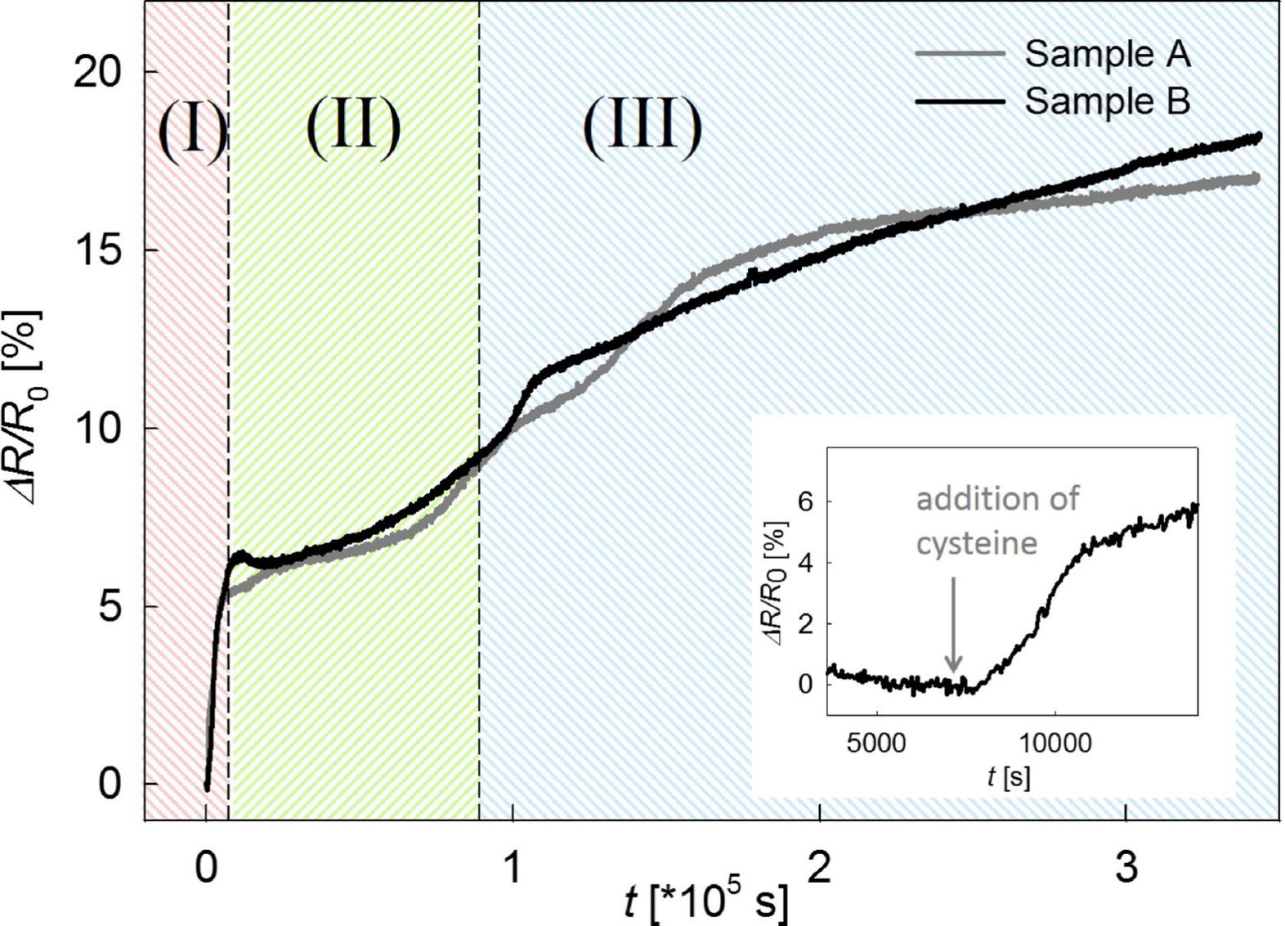
Relative change in electrical resistance measured in situ during the exposure of npAu in 20 mM cysteine solution. Inset: Enlarged first few hours of the resistance change of sample B. After 2 h in ultrapure water, cysteine was added (marked by the grey arrow).

Figure 1 shows the in situ resistance measurement during SAM formation, which yields a total resistance increase of approximately 18%. Both samples exhibit a similar trend, demonstrating the high reproducibility. This overall resistance change is well in line with ex situ studies on npAu in the literature, reporting a relative change in resistance of 6% for propanethiol and 22% for cysteamine monolayer assembly after 24 h [11]. In Figure 1, we can distinguish between three regimes of different slope (denoted as (I) to (III)). Regimes (I) and (III) can be correlated to stages in the adsorption process on planar gold, whereas regime (II) exhibits a behavior unique for nanoporous gold. The steep regime (I) yields an increase of about 4% in about 50 min, which is assigned to cysteine molecules being chemisorbed on the easily accessible outer surfaces. This chemisorption has been reported in the literature to take 30 min for planar Au [7]. Regime (III) shows a very slow increase of the resistance, assigned to reordering of the monolayer, which is known to take several days [12]. After 72 h a total change between 17% and 18% is observed with a fading slope, which yet dropped to less than 1% in 2 days. In the intermediate regime (II), which takes approximately 24 h, the resistance increases by about 5% with a moderate slope. As, to the knowledge of the authors, there is no equivalent stage reported for planar Au [6], we assume this stage to be specific for nanoporosity, representing adsorption on internal surfaces.

In the inset of Figure 1, the first few hours of the resistance change of sample B are shown enlarged. The sample was first stored in water for 2 h prior to the addition of cysteine. Interestingly, the resistance increase attributed to stage (I) starts with a certain delay (about 600 s) after cysteine adding (grey arrow). This delay is attributed to a physisorption step prior to chemisorption (reported to take few minutes for planar Au [13]), which does not significantly change the electronic structure and thus the resistance of the substrate.

**Figure 2:**
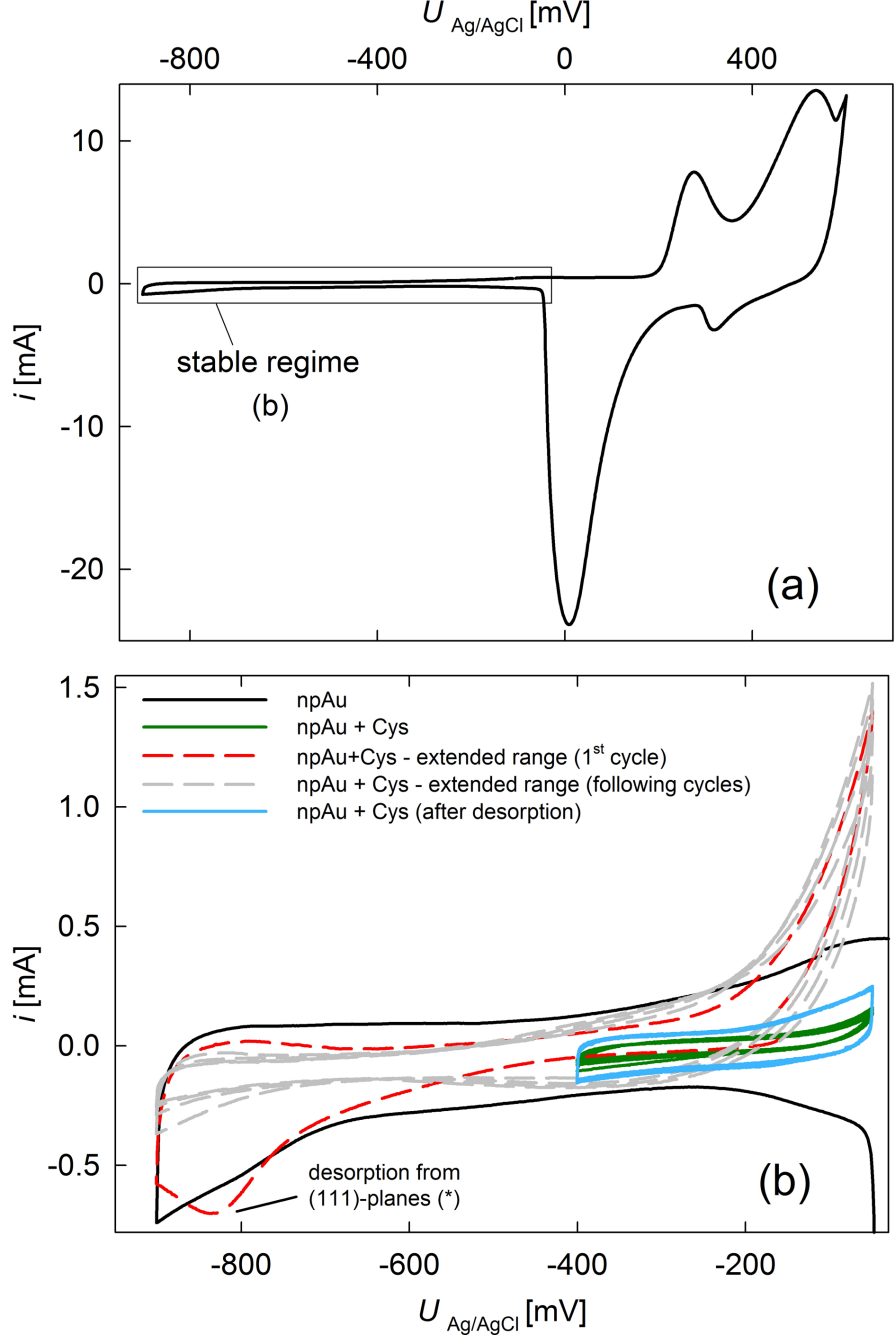
Cyclic voltammetry of sample A. (a) Measurement of the cysteine-free npAu between 600 and −900 mV, (b) CVs recorded after modification with cysteine together with a part of the curve from (a) for reference. In (b), the double layer CV (solid curves) were recorded between −50 and −400 mV and the electrochemical desorption (dashed curves) between −50 and −900 mV. (*)The peak was correlated to the desorption from the (111) plane based on [15]. The blue solid curve was recorded after the dashed curves. All CVs were conducted in 1 M KOH at a scan rate of 1 mV/s. Please note the change in *x*-axis scale between (a) and (b).

Since SAMs are known to decrease the double-layer capacitance of the substrate [14], Figure 2 compares CVs in 1 M KOH of sample A under cysteine-free conditions (before SAM assembly) and after different treatments. The SAMs can be considered stable between −400 mV (SAM desorption) and −50 mV (cysteine oxidation). At −300 mV, a double-layer capacitance of 48 μF/cm^2^ under cysteine-free conditions (Figure 2a,b, black curve) and 8 μF/cm^2^ after cysteine assembly (Figure 2b, solid green curve) can be estimated. When the lower limit of the CV is extended to −900 mV (limited by gas evolution that might cause sample damage), a desorption peak in the first cathodic scan is observed at about −820 mV (Figure 2b, dashed red curve), which does not reappear in the following cycles (Figure 2b, dashed grey curve). For planar Au, several distinct peaks occur during cysteine desorption, which can be assigned to different low-index crystal planes. The weakest bound cysteine molecules desorb at around −700 mV on planar Au, which is attributed to the desorption from the (111) plane [15–16]. It was shown that the desorption of any SAM shifts by about −100 mV when using a nanoporous instead of a planar sample [17]. Thus, based on literature, the cathodic peak at −820 mV in Figure 2b can be attributed to cysteine desorption from the (111) planes of our npAu sample, meaning that the SAM was only partially desorbed. Desorption of stronger bound cysteine was not possible to examine as scanning to even more negative potential would have risked sample damage.

After this desorption peak, significantly higher currents flow near the upper potential limit (Figure 2b, dashed red and grey curves), which are assigned to cysteine oxidation in the solution [18] as the desorbed cysteine is now dissolved in the electrolyte. The double-layer capacitance now amounts to 20 μF/cm^2^ (Figure 2b, solid blue curve), which is still significantly lower than the initial value.

**Figure 3:**
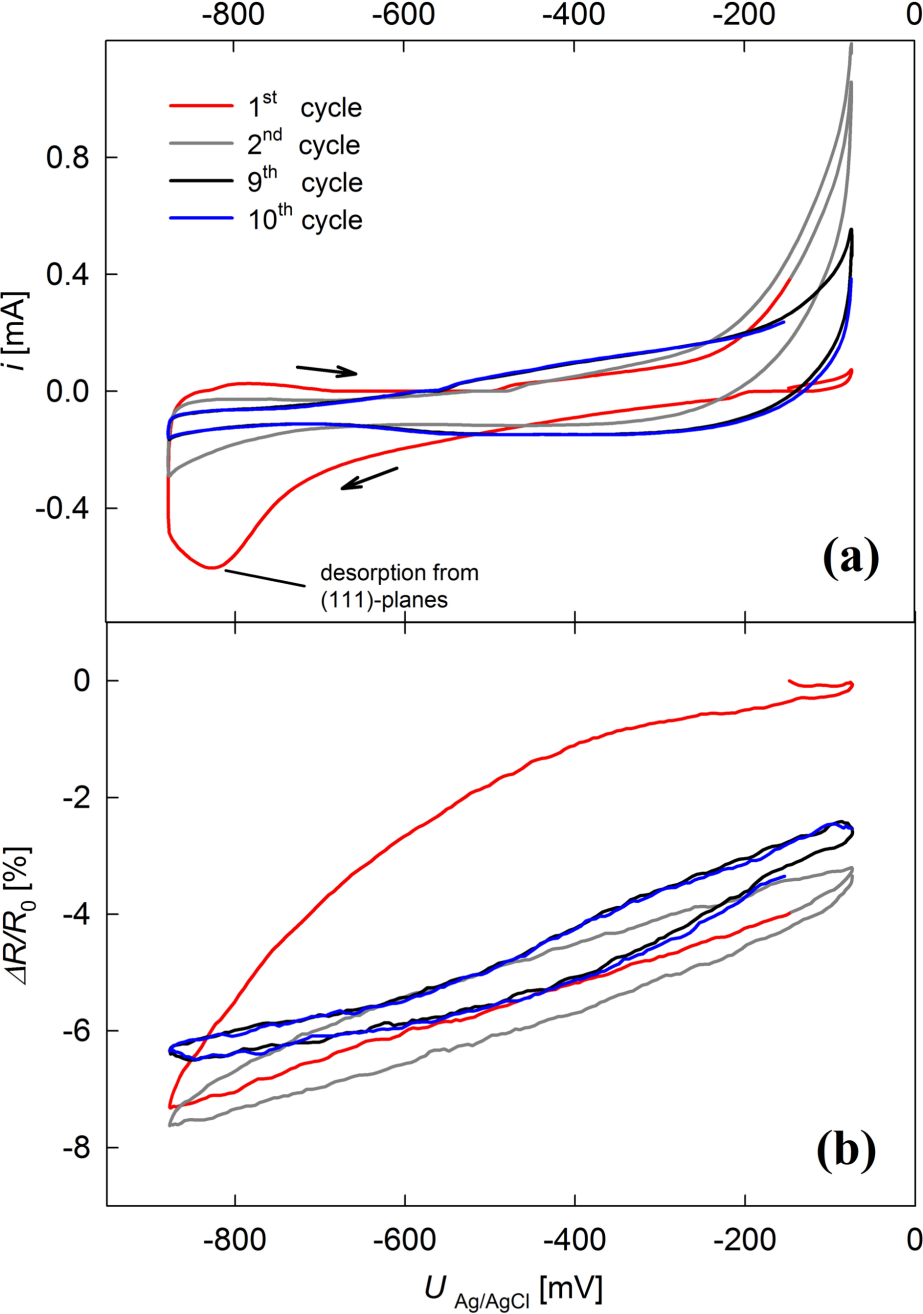
Cyclic voltammetry of sample B modified with cysteine (a) and concomitantly measured change in relative electrical resistance (b). The potential was varied between −900 and −50 mV with a scan rate of 1 mV/s in 1 M KOH.

The relative change in electrical resistance during cysteine desorption from the (111) planes and subsequent cycling between −900 and −50 mV was monitored in situ for sample B, as shown in Figure 3. It demonstrates good reproducibility of the desorption peak at −820 mV.

During desorption, the resistance (Figure 3b) strongly decreases by about 7.2%. In subsequent cycles it varies in a range of 4%, matching our earlier results for double-layer charging of npAu [19]. A slight drift during repeated cycling is most likely caused by sample degradation.

The charge transfer of 0.15 C during desorption is a superposition of actual cysteine desorption and double-layer charging (with an increasing contribution as cysteine is desorbed). When we assume the double-layer capacitance to increase roughly proportionally to the total charge transfer, this yields contributions of 0.06 C for the double layer and 0.09 C for the cysteine desorption. This charge transfer can be associated with a resistance variation of 2.4% (estimated from the variations upon double-layer charging in following cycles) and 4.8%, respectively. Assuming equal contributions of desorption from different crystallographic planes to the resistance change and a transfer of one electron per cysteine molecule, the total amount of cysteine (3.5 × 10^−6^ mol) on the npAu can be deduced from the initial resistance increase of 18% during the assembly. With a specific surface area of 10 m^2^/g (determined following [20]), an initial surface coverage of 0.48 monolayers can be estimated. This value agrees well with SAM surface coverages reported in the literature [21–23].

The sensitivity of resistance measurements towards ad-/desorption can be expressed by the charge coefficient, which relates the relative resistance variations to the imposed charge per mole. Here, a value of 9.1 × 10^−5^ mol/C is obtained for cysteine, which is approximately twice as high as that obtained for the ad-/desorption of oxygen on npAu [19], approving this technique to be suitable for sensitively detecting even small amounts of SAMs on npAu.

In conclusion, in situ resistometry proved to be a highly sensitive tool for dynamically monitoring the formation process of self-assembled monolayers, exemplarily using cysteine. The assembly takes place with a total resistance increase of about 18% in three clearly distinguishable regimes. The three regimes can be assigned to the adsorption on easily accessible outer surfaces, followed by adsorption on internal surfaces of the nanoporous structure and finally a reordering stage, taking several days. In cyclic voltammetry, a desorption of the SAM could be achieved from the crystallographic (111) planes, along with a resistance decrease of 4.8%, determined by in situ resistometry. From this, an initial surface coverage of 0.48 cysteine monolayers could be estimated. As an outlook, influencing parameters such as the cysteine concentration in solution, which would affect the time period of the adsorption [24], and the pH value of the solution, which would impact the stability of the SAMs [25], could be examined further.

## References

[1] Xiao X, Li H, Wang M, Zhang K, Si P (2014). Analyst.

[2] Meng F, Yan X, Liu J, Gu J, Zou Z (2011). Electrochim Acta.

[3] Lang X Y, Yuan H T, Iwasa Y, Chen M W (2011). Scr Mater.

[4] Xiao X, Si P, Magner E (2016). Bioelectrochemistry.

[5] Mameka N, Lührs L, Heissler S, Gliemann H, Wöll C (2018). ACS Appl Nano Mater.

[6] Vericat C, Vela M E, Benitez G, Carro P, Salvarezza R C (2010). Chem Soc Rev.

[7] Qingwen L, Hong G, Yiming W, Guoan L, Jie M (2001). Electroanalysis.

[8] Hakamada M, Takahashi M, Furukawa T, Tajima K, Yoshimura K, Chino Y, Mabuchi M (2011). Phys Chem Chem Phys.

[9] Steyskal E-M, Qi Z, Pölt P, Albu M, Weissmüller J, Würschum R (2016). Langmuir.

[10] Jin H-J, Parida S, Kramer D, Weissmüller J (2008). Surf Sci.

[11] Hakamada M, Kato N, Mabuchi M (2016). Appl Surf Sci.

[12] Sumi T, Wano H, Uosaki K (2003). J Electroanal Chem.

[13] Torrelles X, Vericat C, Vela M E, Fonticelli M H, Daza Millone M A, Felici R, Lee T-L, Zegenhagen J, Muñoz G, Martín-Gago J A (2006). J Phys Chem B.

[14] Campuzano S, Pedrero M, Montemayor C, Fatás E, Pingarrón J M (2006). J Electroanal Chem.

[15] Arihara K, Ariga T, Takashima N, Arihara K, Okajima T, Kitamura F, Tokuda K, Ohsaka T (2003). Phys Chem Chem Phys.

[16] Huang J-F, Sun I-W (2005). Adv Funct Mater.

[17] Chu Y, Seo B-R, Kim J-W (2010). Bull Korean Chem Soc.

[18] Fawcett W R, Fedurco M, Kováčová Z, Borkowska Z (1994). J Electroanal Chem.

[19] Wahl P, Traußnig T, Landgraf S, Jin H-J, Weissmüller J, Würschum R (2010). J Appl Phys.

[20] Lakshmanan C, Viswanath R, Polaki S, Rajaraman R (2015). AIP Conf Proc.

[21] Widrig C A, Chung C, Porter M D (1991). J Electroanal Chem Interfacial Electrochem.

[22] Love J C, Estroff L A, Kriebel J K, Nuzzo R G, Whitesides G M (2005). Chem Rev.

[23] Cortés E, Rubert A A, Benitez G, Carro P, Vela M E, Salvarezza R C (2009). Langmuir.

[24] Han Y, Uosaki K (2008). Electrochim Acta.

[25] Kong B-K, Kim Y-S, Choi I S (2008). Bull Korean Chem Soc.

